# Dietary Patterns, Oxidative Stress, and Early Inflammation: A Systematic Review and Meta-Analysis Comparing Mediterranean, Vegan, and Vegetarian Diets

**DOI:** 10.3390/nu17030548

**Published:** 2025-01-31

**Authors:** Sara Ilari, Stefania Proietti, Francesca Milani, Laura Vitiello, Carolina Muscoli, Patrizia Russo, Stefano Bonassi

**Affiliations:** 1 Department of Human Sciences and Quality of Life Promotion, San Raffaele University, 00166 Rome, Italy; sara.ilari@sanraffaele.it (S.I.); francesca.milani@uniroma5.it (F.M.); laura.vitiello@uniroma5.it (L.V.); stefano.bonassi@uniroma5.it (S.B.); 2 Pain Physiology and Pharmacology, IRCCS San Raffaele Roma, 00166 Rome, Italy; 3 Agea, Coordinating Body, 00185 Rome, Italy; s.proietti@agea.gov.it; 4 Clinical and Molecular Epidemiology, IRCCS San Raffaele Roma, Via di Val Cannuta 247, 00166 Rome, Italy; 5 Department of Health Science, Institute of Research for Food Safety and Health (IRC-FSH), University “Magna Graecia” of Catanzaro, 88100 Catanzaro, Italy; muscoli@unicz.it

**Keywords:** oxidative stress, inflammation, Mediterranean diet, vegetarian diet, vegan diet, plant-based diet, healthy subject

## Abstract

Background: Dietary habits influenced by lifestyle and cultural factors play a critical role in health by modulating oxidative stress and inflammation. While diets offer significant benefits, they may also pose risks, such as nutrient deficiencies, emphasizing the need for a balanced approach. Exploring Mediterranean and plant-based diet effects on oxidative stress and inflammation biomarkers may help improve health outcomes and disease prevention strategies. Methods: This study analyzed 65 studies following PRISMA guidelines to evaluate the effects of Mediterranean and plant-based diets on biomarkers of oxidative stress and inflammation in healthy individuals. Results: The Mediterranean diet was weakly associated with reductions in oxidative stress markers, including MDA (ROM: 0.80; 95% CI: 0.57–1.13; *p* = 0.2092) and 8OHdG (ROM: 0.81; 95% CI: 0.59–1.11; *p* = 0.1847), as well as inflammation markers such as CRP (ROM: 0.72; 95% CI: 0.42–1.23; *p* = 0.1545) and IL-6 (ROM: 1.23; 95% CI: 0.97–1.55; *p* = 0.08). The vegetarian diet significantly reduced CRP (ROM: 0.82; 95% CI: 0.69–0.98; *p* = 0.0297), while the vegan diet showed a borderline reduction (ROM: 0.81; 95% CI: 0.56–1.17; *p* = 0.2544), suggesting lower systemic inflammation compared to omnivorous diets. Conclusions: Although all three diets demonstrate potential in reducing oxidative stress and inflammation, the antioxidant effects—especially for the Mediterranean diet—are lower than anticipated, indicating alternative mechanisms. Further research is essential to confirm these findings and clarify the underlying mechanisms to enhance preventive health strategies.

## 1. Introduction

Dietary habits, influenced by lifestyle traditions, socio-demographic characteristics, and ethnicity, have a direct impact on people’s wellness and quality of life. Although specific foods and nutrients play a significative role in keeping good health, it has become clear that overall dietary patterns are more closely linked to well-being and health [1].

Nutrition is a key factor in regulating reactive oxygen species (ROS) levels and the degree of inflammation in the body. Some foods act as protective shields, rich in antioxidants and nutrients that help to reduce these harmful processes [1]. Diets such as the Mediterranean and plant-based ones are particularly effective in modulating these factors due to their content of antioxidants and anti-inflammatory agents [1].

Among these, the Mediterranean diet stands out for its health benefits. It is characterized by a high intake of vegetables, legumes, fruits, and cereals, with white meat and fish as primary protein sources and olive oil as the main fat source [2,3]. Numerous epidemiological studies show that the adherence to this diet is associated with a significant reduction in mortality and cardiovascular diseases [2,3]. Similarly, plant-based diets, such as vegetarian and vegan diets, are associated with health benefits like lower consumption of saturated fats and higher intake of dietary fiber [4]. However, there are also potential risks, such as reduction in vitamin, vitamin B12, calcium, and iodine, particularly for those following a vegan diet [4].

In this regard, studies found that vegetarians have a relatively low risk of developing ischemic heart disease, diabetes, kidney stones, diverticulitis, and cancer compared to meat-eaters [4]. However, these individuals showed a relatively higher risk of stroke, particularly hemorrhagic stroke, and bone fractures [4]. For vegans, the same study indicated a lower risk of diabetes, cataracts, and diverticulitis but a higher risk of fractures [4]. The data currently available are insufficient to draw definitive conclusions [4].

Understanding how diet modulates oxidative stress and inflammation is crucial for developing dietary strategies that enhance quality of life and lower disease risk. Disease development and aging are deeply influenced by oxidative stress [5,6]. Free radicals, primarily ROS and reactive nitrogen species (RNS), are naturally produced during metabolic processes. While ROS and RNS are essential for normal cellular functions, excessive levels can cause oxidative stress, altering DNA, proteins, and lipids [7]. This damage is linked to various diseases, including cardiovascular diseases, cancer, lung diseases, neurological disorders such as Alzheimer’s disease, and other disorders characterized by multiple mechanisms through which ROS cause cellular damage [8]. Biomarkers of oxidative stress, e.g., malondialdehyde (MDA) and 8-hydroxydeoxyguanosine (8OHdG), are often used to assess the extent of cellular damage [8,9,10].

Oxidative stress is closely linked to inflammation. In fact, the accumulation of ROS activates inflammatory pathways, stimulating the production of pro-inflammatory cytokines such as IL-6 and TNF-α [11]. This process contributes to the development of chronic inflammation, which is a long-term response to oxidative stress eventually leading to tissue damage [11]. When the inflammatory process becomes chronic, as seen in autoimmune, cardiovascular, or neurodegenerative diseases, a vicious cycle is triggered: chronic inflammation further promotes ROS production, which intensifies cellular damage and exacerbates the inflammatory state [8,9,10,11].

The inflammatory response is regulated by a wide range of mediators that act as the main agents of inflammation, with many of them influencing the vascular system and facilitating the recruitment of leukocytes. Most studies have assessed C-reactive protein (CRP) as the primary indicator of the effects of a vegetarian diet, while a smaller number of studies have examined IL-6, tumor necrosis factor-alpha (TNF-α), adiponectin, and IL-10 [11].

CRP is part of the pentraxin superfamily and is well-known as a key indicator of inflammation and cardiovascular risk in humans. Recent research highlights that CRP not only serves as a marker of inflammation but also plays an active role in triggering inflammatory responses and supporting the innate immune system [12,13]. CRP is an acute-phase protein mainly produced and secreted by the liver. In response to injury or infection, CRP levels in the bloodstream can surge from baseline values of less than 1 μg/mL within 48 h. Additionally, CRP concentrations increase in chronic inflammatory conditions, such as cardiovascular and autoimmune diseases. Due to its strong link with inflammation, CRP has gained widespread attention as a non-specific marker for tracking and assessing infections and inflammation as well as a prognostic tool for predicting cardiovascular events. However, new evidence indicates that CRP not only reflects inflammation but also actively regulates innate immunity and the progression of inflammatory responses [14].

Pro-inflammatory cytokines, namely interleukins (IL-1β, IL-6, IL-12, and IL-18), along with interferon-γ (IFN-γ) and tumor necrosis factor-alpha (TNF-α), are secreted by immune cells. Increased levels of IL-6, TNF, and IL-1β have been linked to major non-communicable diseases, disability, and higher mortality rates in older adults [15].

In aerobic organisms, the continuous production of free radicals necessitates a balance, maintained by antioxidants. These substances reduce the formation of free radicals and neutralize or repair the induced damage. Both endogenous and exogenous antioxidants play crucial roles in the reaction to oxidative damage and inflammation [16]. Enzymatic antioxidants, such as superoxide dismutase (SOD), glutathione peroxidase (GPX), peroxiredoxin (PRDx), and catalase (CAT), play a primary role in neutralizing harmful ROS [16].

Generally, natural antioxidants are primarily obtained from the diet; these include small molecules such as vitamins C and E, carotenoids, and flavonoids (polyphenols). Experimental evidence in vitro and ex vivo (animals) demonstrated that polyphenols defend cellular components from oxidative damage and help prevent degenerative diseases linked to oxidative stress [17,18,19,20]. Plant polyphenols can reduce the impact of cytokines by modulating their receptors or diminishing their secretion processes [21]. Phenolic compounds, by acting on NF-kB, can block COX-2 and NO synthase functions, preventing the activation of immune cells. Phenolic acid can decrease the production of IL-6, TNF-α, and IL-1β at both the gene and protein levels.

The effect of antioxidant defense depends upon the role oxidative stress plays in the pathogenesis [17]. When oxidative stress assumes an auxiliary role in the disease, which occurs more frequently, damage mitigation might not significantly impact the progression of the pathological condition. This is one of the primary reasons why antioxidants often fail to exert a significant effect on human pathology, despite the evident reduction in oxidative stress markers [17]. This limitation may be the most important factor frequently underestimated in clinical trials on antioxidant treatment [21].

The modern lifestyle, marked by poor dietary habits, physical inactivity, and exposure to chemicals such as heavy metals, food additives, and pesticides, contributes to priming the oxidative stress, possibly increasing the frequency of chronic diseases, as reported by numerous experimental and clinical studies [21].

In summary, while the Mediterranean and plant-based diets show promise in promoting long-term health by reducing oxidative stress and inflammation, further research is needed to fully understand their impact, particularly in clinical contexts. For this reason, the aim of this systematic review is to evaluate the impact of the main diets used as a lifestyle, such as the Mediterranean and plant-based diets (vegetarian and vegan diets), on the modulation of oxidative stress and early-stage inflammation markers.

## 2. Materials and Methods

### 2.1. Literature Search

The systematic review identified 65 papers, which were divided into two groups based on the type of diet (Mediterranean or plant-based); both included a meta-analysis focusing on specific oxidative stress markers (8OHdG, MDA) and early inflammatory markers (CRP, IL-6, TNF-α).

We used the following search terms: “genomic stability” OR “DNA damage” OR “oxidative stress” OR ”8OHdG” OR “MDA OR malondialdehyde” OR “C-reactive protein OR CRP” OR “IL-6” OR “TNFα” OR “GSH” OR “GPx” OR “IL10” OR “inflammation” OR “early-stage inflammation”, associated to the search terms “diet”, “mediterranean diet”, “vegetarian diet”, vegan died”, “plant-based diet”.

Studies included in this systematic review were retrieved from PubMed, MEDLINE, the Cochrane Library, and Web of Science databases, adhering to the PRISMA (Preferred Reporting Items for Systematic Reviews and Meta-Analyses) guidelines and following the PICOs framework (Population, Intervention, Comparison, Outcome, Population). We evaluated all papers written in English and published up to June 2024. Additionally, we examined the reference list of all the included papers in order to verify the presence of other eligible studies that were not indexed in the databases mentioned above.

### 2.2. Study Selection

The analysis included studies that met the following criteria: human studies on subjects following a Mediterranean, vegan, or vegetarian diet compared to a control group (generally omnivorous) in relation to markers of oxidative stress or early inflammation. Exclusion criteria: (1) Studies not published in the English language; (2) conference abstracts, editorials, book series, errata, or conference proceedings; (3) incomplete studies; (4) animal or cellular models; (5) studies analyzing the consumption of single foods, supplements, or natural antioxidants; and (6) studies without quantitative data on markers of oxidative stress or inflammation.

### 2.3. Data Extraction and Quality Assessment

The primary objective of this study was to evaluate the effectiveness of selected diets as lifestyle factors in mitigating oxidative stress, DNA damage, and early-stage inflammation in healthy individuals. To achieve this aim, we focused on key biomarkers that have been widely studied in recent years in the literature. Based on this review, we selected and analyzed the following biomarkers: MDA (malondialdehyde), DNA damage (measured using the comet assay), oxidative stress-induced DNA damage (indicated by the presence of 8OHdG), GSH, GPx, CRP (C-reactive protein), IL-6, and TNF-α.

Based on the data retrieved from the literature search, we conducted several meta-analyses to assess the effect of dietary habit on selected biomarkers. We focused on those regimens for which sufficient data were available. The analyses compared the effects of the Mediterranean and plant-based diets (vegan or vegetarian; experimental group) against an omnivorous biomarker; combinations lacking sufficient data were treated in line with systematic review principles; and the available evidence was described on a case-by-case basis.

### 2.4. Data Synthesis and Analysis

The association between dietary regimen and specific biomarkers was summarized in three groups, and results were reported by each dietary regimen. DerSimonian and Lard random effects model was used to calculate weighted means and their corresponding 95% confidence intervals (CIs). The effect of each dietary regimen included in the meta-analyses on selected biomarkers was quantified using the ratio of means (ROM) for association. This measure of effect is independent from the absolute values of the means, reduces the extent of interlaboratory variability, and increases the comparability across the studies considered. The presence of heterogeneity among studies was tested with Cochran’s Q test and I^2^ statistics [22]. Publication bias was assessed by visual inspection of funnel plots and testing the asymmetry of the plot with the Egger’s test. Meta-analyses were performed in R 4.4.0 using the metaphor, meta package. Primary outcomes relating to variance and ratio of means were pooled across studies using random effects models. To check the stability of the estimated measures, a sensitivity analysis was conducted using “leave-one-out” procedure when the number of the studies was >3 with the function metainf. All statistical tests were carried out at a two-tailed alpha-level of <0.05 [23].

## 3. Results

### 3.1. Analysis of the Study Selection

**Figure 1:**
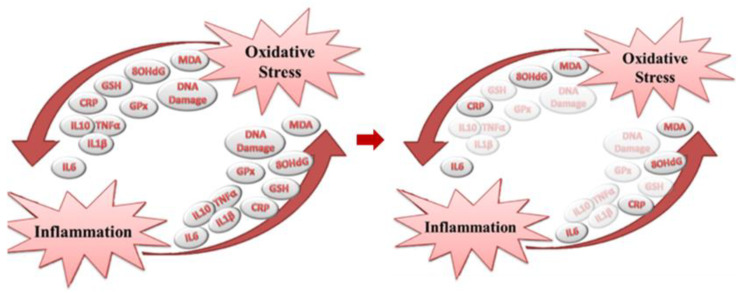
Biomarker involved in the development of oxidative stress and early-stage inflammation (**left panel**), and factors included in the systematic review and meta-analysis (**right panel**).

Analysis was initially conducted by considering the major biomarkers of oxidative stress and the factors involved in early-stage inflammation. The systematic review identified 8588 records from the literature search. We excluded 976 papers as duplicates and 3146 because their contents did not align with the aims of the review. A total of 4466 articles were assessed for eligibility, and 1415 were excluded as they were either review, in vitro studies, congress communications, or lacked complete dates. A total of 41 experimental studies were included in the qualitative analysis, comprising 65 markers in total: 8OHdG (*n* = 7), MDA (*n* = 9), comet assay (*n* = 4), C-reactive protein (*n* = 25), IL-6 (*n* = 8), TNF-α (*n* = 7), and GSH (*n* = 5). Those studies that could not be included in the meta-analysis for specific diet/biomarkers association were individually commented as in a systematic review (Figure 1).

**Figure 2:**
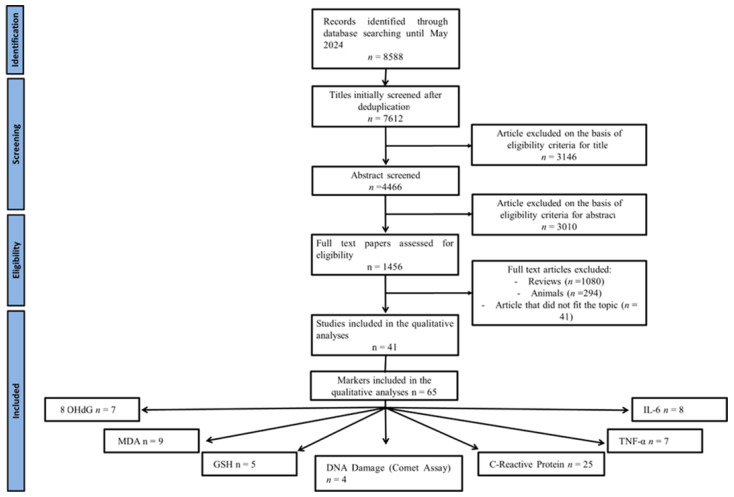
PRISMA flow chart of the literature search.

Figure 2 illustrates the process of the literature search and papers screening (PRISMA flow chart).

### 3.2. Study Characteristics: Systematic Review of Oxidative and Inflammation Markers in Mediterranean Diet (MedDiet)

Table 1 presents a summary of the literature search on MedDiet, reporting main features of the studies selected and its effect on markers of oxidative (MDA, 8OHdG, and DNA damage via comet assay, GSH) and early-stage inflammation (CRP, IL-6, TNF-α).

Numerous studies reported changes in oxidative and inflammatory markers in relation to dietary lifestyle [24,25,26]. Among them, the study of Azzini and colleagues showed that subjects following a diet characterized by a higher consumption of fruits and vegetables are protected from lipid oxidation due to oxidative stress. In this regard, lower levels of MDA are observed in individuals with medium or high adherence to the MedDiet compared to those with low adherence; although this result is not statistically significant [25]. Similarly, the study conducted by Quetglas-Llabrés and colleagues [26] evaluated the effects of MedDiet on the level of oxidative stress and inflammation in individuals at high risk of cardiovascular diseases over a period of 2 years. The results of this study showed lower levels of plasma MDA in patients after a 2-year follow-up compared to baseline values. This decrease may be favored by an increase in circulating polyphenols, associated with better adherence to the Mediterranean diet. The study also analyzed the levels of 8OHdG in urine: a decrease in 8OHdG levels was observed after two years of MedDiet, while MDA levels remained stable. However, the results of the analysis were not statistically significant. As regards inflammation, no pre-/post-differences were found in CRP levels in subjects starting a MedDiet regimen. In contrast, a pronounced decrease in IL-6 levels was observed [26]. The study of Mesquida and colleagues reported that participants with metabolic syndrome who had high adherence to the MedDiet showed better inflammatory and oxidative status compared to metabolic syndrome patients with low adherence to the diet. No differences in MDA levels were observed based on adherence to the MedDiet, in keeping with the absence of differences in urinary MDA and in the ratio of 8OHdG/creatinine [27]. Similar results were observed in the Parcina et al. study, where no significant changes in oxidative stress markers were observed in patients following the MedDiet compared to control subjects, except for a reduction in MDA levels [28]. Thompson and colleagues observed that urinary MDA is a less sensitive marker for detecting differences in peroxidation in subjects where the number of fruits and vegetables in the Mediterranean diet was increased. Additionally, the dietary intervention reduced lymphocytic levels of 8OHdG in subjects with low pre-intervention plasma levels of alpha-carotene, but not in those with high levels. This result is important because it indicates that increasing intake of fruits and vegetables may reduce DNA oxidation [29]. A significant reduction in 8OHdG levels was reported by Mitjavila et al. in patients who increased their intake of extra virgin olive oil in the Mediterranean diet compared to subjects on a classic Mediterranean diet [30]. Finally, Yeon et al. [31] observed that following a MedDiet enriched in fruits and vegetables led to a significant reduction in DNA damage, as measured by the comet assay. These results are supported by previous studies indicating that a diet rich in carotenoids and polyphenols has protective effects against DNA damage [31]. Measurements of 8OHdG did not show significant differences between the different diets, suggesting that other factors might influence the measure of 8OHdG as a marker of oxidative stress. From an inflammatory perspective, a decrease in IL-6 was observed across different diets, although it was not statistically significant [31].

The only dietary intervention that showed an effect in preventing major cardiovascular events in a randomized, controlled trial was found to be the Mediterranean diet supplemented with olive oil or nuts. Those studies [32,33] showed a significant difference between CRP levels in the subjects randomized to the MedDiet. On the contrary, in the study by Murphy et al., no changes were observed between CRP levels in 18 months of MedDiet. These data were confirmed in the study by Quetglas-Llabrés et al. [24], in which no differences in CRP levels were found after starting MedDiet regimen. Compared to the control group, participants in the MedDiet group showed no significant changes in any inflammatory biomarkers, including IL-6 [32].

**Table 1:** Markers of oxidative and early-stage inflammation and their impact on Mediterranean diet in healthy subjects.

Study, Year	Type of Study	Population	Mean, Age, Years (SD/IC)	Sex (M/F)	Dietary Pattern/Dietary Assessment Method	Biomarkers	Results	*p* Value
Quetglas-Llabrés et al., 2024 [24]	Randomized trial	Mediterranean pre and post *n* = 20	Range 48 to 60 years	Mediterranean pre and post % (50 M/50 F)	Mediterranean Adherence to diet assess by MDP	CRP	Mediterranean Pre 0.4 ± 0.5 Post 0.4 ± 0.4	NS
Azzini et al., 2011 [25]	Observational study	Mediterrean diet *n* = 131	20–40 years	64 M/67 F	Mediterranean diet (low, medium, high) Adherence to diet assess by MDS	MDA TNF-alpha	MDA (uM) Low 118 ± 7 Medium 104 ± 7 High 110 ± 7 TNF-alpha Low 42.4 ± 9.0 Medium 35.7 ± 7.0 High 14.8 ± 2.9	NS 0.05
Quetglas-Llabrés et al., 2022 [26]	Prospective randomized trial	Mediterranean diet high adherence *n* = 45 Mediterranean diet low adherence *n* = 45	High adherence 64.2 ± 0.4 Low adherence 64.2 ± 0.4	NA	Mediterranean diet (low and high adherence) Adherence to diet assess by MDP	8OHdG urine MDA plasma IL-6TNF-alpha	8OHdG High 1.93 ± 0.06 Low 2.01 ± 0.05 MDA (nM) High 0.928 ± 0.066 Low 0.922 ± 0.071 IL-6 High 3.09 ± 0.41 Low 6.71 ± 0.71 TNF-alpha High 56.3 ± 2.9 Low 67.8 ± 4.4	0.244 0.821 NS 0.015
Monserrat-Mesquida et al., 2022 [27]	Prospective cohort analysis of data obtained between baseline and 2-year parallel group of a randomized trial	Control group *n* = 49 Intervention diet group *n* = 48 (baseline and 2 years after)	Control group 64.5 ± 0.5 Intervention diet 64.9 ± 0.4 (baseline and 2 years after)	NA	Mediterranenan diet Adherence to diet assess by MDS	8OHdG urine MDA Urine and plasma	**8OHdG** Control 1.49 ± 0.092 Diet 1.17 ± 0.051 **MDA plasma** Control 1.190 ± 0.085 Diet 0.467 ± 0.035 **MDA urine** Control 94.4 ± 10.8 Diet 111.0 ± 12.5	0.247 <0.001 0.652
Parcina et al., 2015 [28]	NA	Baseline *n* = 39 Mediterranean diet *n* = 14	Baseline 9.5 ± 5.9 Mediterranean diet 31.88 ± 6.25	Baseline (39 M) Mediterranean diet (14 M)	Mediterranean diet Adherence to diet: not reported	8OHdG, MDA	**8OHdG** Baseline 66.21 ± 12.4 Mediterrean diet 66.96 ± 16.0 **MDA** Baseline 13.85 ± 7.13 Mediterrean diet 7.598 ± 5.699	NS NS
Thompson et al., 1999 [29]	NA	Mediterranean diet (pre-interventions) *n* = 28 Mediterranean diet (post-interventions) *n* = 28	49.8 years, range 27–80	Mediterranean diet (pre-interventions) (28 F) Mediterranean diet (post-interventions) (28 F)	Mediterranean diet Adherence to diet: not reported	8OHdG, MDA	8OHdG Urine ng/mL Pre 49.6 ± 12.4 Post 21.4 ± 2.2 Lymphocytes ng/mg Pre 7.9 ± 1.2 Post 6.2 ± 0.8 MDA urine nmol/mg Pre 11.7 ± 0.8 Post 11.7 ± 1	NS NS NS
Mitjavila et al., 2013 [30]	Randomized controlled trial	(1) Mediterranean diet + OLIO EVO *n* = 38 (2) Mediterranean diet + nuts *n* = 35 (3) Control diet *n* = 37	Mediterranean diet + EVO (69.2 ± 5.7) Mediterranean diet + nuts (68.6 ± 5.2) Control diet (68.2 ± 5.3)	Mediterranean diet + EVO (38 F) Mediterranean diet + nuts (35 F) Control diet (37 F)	Mediterranean diet/Control diet Adherence to diet assess by semi-quantitative fodo frequency questionnaire	8OHdG	Baseline (1) 20.24 (0.75) (2) 19.98 (0.77) (3) 22.82 (0.73) Follow-up: (1) 11.19 (0.59) (2) 9.78 (0.61) (3) 19.95 (0.58)	0.013; <0.001
Yeon et al., 2012 [31]	NA	(1) Mediterranean diet with high consumption of fruits and vegetables *n* = 102 (2) Mediterranean diet with low consumption of fruits and vegetables *n* = 102	23.14 ± 3.06	(102 F)	Mediterranean diet Adherence to diet: not reported	8OHdG, Comet assay IL-6	8OHdG: (1) Pre 0.31 ± 0.02 Post 0.32 ± 0.02 (2) Pre 0.32 ± 0.04 Post 0.31 ± 0.02 Comet assay: tail moment (%) Pre 5.70 ± 2.48 Post 3.75 ± 1.47 Tail length (%) Pre 53.26 ± 13.18 Post 32.84 ± 12.06 IL-6 Pre 3.52 ± 1.08 Post 3.44 ± 0.83	0.55 0.0080 0.0001 NS
Jaacks et al., 2018 [32]	Randomized controlled trial	Mediterranean *n* = 9 Control *n* = 11	Mediterranean 51.4 ± 6.6 Control 51.4 ± 6.6	Mediterranean and Control % (26.7 M/73.3 F)	Mediterranean/control Adherence to diet: not reported	CRP IL-6 GSH	CRP Mediterranean 1.76 ± 1.72 Control 5.22 ± 5.73 IL-6 Mediterranean 1.30 ± 1.23 Control 2.03 ± 2.37 GSH Mediterranean 51.4 ± 6.6 Control 51.4 ± 6.6	NS NS NS
Murphy et al., 2022 [33]	Randomized parallel control study	Mediterranean *n* = 55 Habit diet *n* = 53	Mediterranean 71.0 ± 4.9 Habit diet 70.9 ± 4.9	Mediterranean % (42.5 M/57.5 F) Habit diet % (44.9 M/55.1 F)	Mediterranean diet (low and high adherence) Adherence to diet assess by MDP	CRP	Mediterranean 1.44 ± 0.26 Habit diet 1.69 ± 0.30	NS
Pagliai et al., 2024 [34]	Randomized, clinical trial	Mediterranean diet *n* = 27 Vegetarian diet *n* = 25	Mediterranean diet 48.11 ± 13.43 Vegetarian diet 50.24 ± 11.37	Mediterranean diet (7 M/20 F) Vegetarian diet (6 M/19 F)	Mediterranean diet/Vegetarian diet Adherence to diet assess by modified version of the National Health and Nutrition Examination Survey food questionnaire	IL-6 TNF-alpha	IL-6 Mediterranean diet 1.06 (0.83–1.28) Vegetarian diet 1.09 (0.82–1.35) TNF-alpha Mediterranean diet 4.32 Vegetarian diet 4.38	NS NS

MDS, Mediterranean Diet Score; and MDP, Mediterranean Diet Pyramid questionnaire.

### 3.3. Meta-Analysis of Oxidative and Inflammation Markers in MedDiet

**Figure 3:**
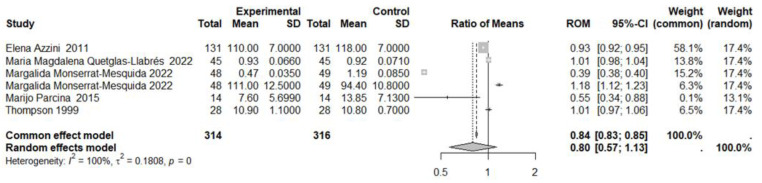
Forest plot showing the effects of MedDiet on MDA. SD, standard deviation; ROM, ratio of means; and CI, confidence interval [25,26,27,28,29].

In the first meta-analysis, six studies on MDA value were considered, with a total of 314 subjects in the experimental group (MedDiet) and 316 subjects in the control group (Figure 3). High heterogeneity between studies was observed: I^2^ = 100%, *p* = 0 (Figure 3). Results generated by random effects model showed no statistically significant reduction in the experimental group, compared to the control group (ROM: 0.80; 95% CI: 0.57 to 1.13; *p* = 0.2092). Visual inspection of the funnel plot did not identify substantial asymmetry, and the lack of publican bias was confirmed by the Egger’s test (t = −0.16, *p* = 0.882 bias estimate −3.35 (SE = 21.2)). The sensitivity analysis conducted using “leave-one-out” procedure confirmed that there was no statistically significant reduction in the experimental group, compared to the control group.

**Figure 4:**
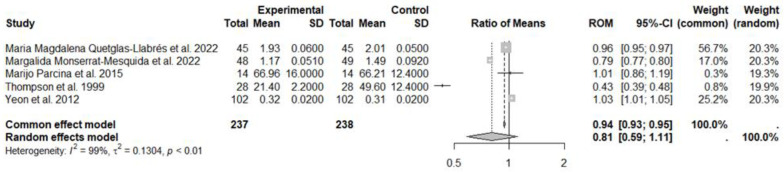
Forest plot showing the effects of MedDiet on 8OHdG. SD, standard deviation; ROM, ratio of means; and CI, confidence interval [26,27,28,29,31].

In the second meta-analysis, five studies on 8OHdG values were considered, with a total of 237 subjects in the experimental group (MedDiet) and 238 subjects in the control group (Figure 3). The meta-ROM generated by random effects model showed no statistical reduction in this biomarker in the experimental group, compared to the control group (ROM: 0.81; 95% CI: 0.59 to 1.11; *p* = 0.1847). High heterogeneity between studies was observed: I^2^ = 99%, *p* < 0.01 (Figure 4). No publication bias was suggested by the visual inspection of the funnel plot or by the Egger’s test (t = −1.01, *p* = 0.338 bias estimate −9.62 (SE = 9.6)). The sensitivity analysis conducted using “leave-one-out” procedure confirmed that there was no statistically significant reduction in the experimental group, compared to the control group.

**Figure 5:**
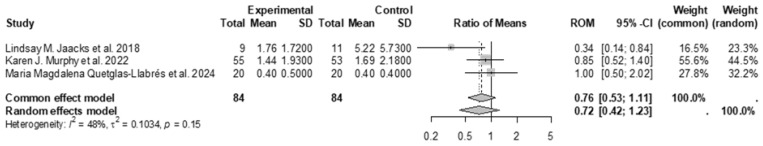
Forest plot showing the effects of MedDiet on CRP. SD, standard deviation; ROM, ratio of means; and CI, confidence interval [24,32,33].

In the third meta-analysis, three studies on CRP values were included, with a total of 84 subjects in the experimental group (MedDiet) and 84 subjects in the control (Figure 5). Also, for these biomarkers, no significant changes were observed between groups. Results generated by common effect model showed no statistically significant improvements in the experimental group (ROM: 0.76; 95% CI: 0.53 to 1.11; *p* = 0.1545). Low heterogeneity between studies was observed: I^2^ = 48%, *p* = 0.15 (Figure 5), and no evidence of publication bias was found (Egger’s test t = −0.99, *p* = 0.503 bias estimate −3.28 (SE = 3.3)).

**Figure 6:**
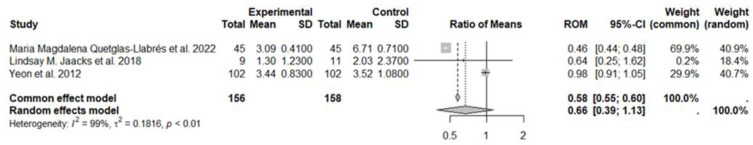
Forest plot showing the effects of MedDiet on IL-6. SD, standard deviation; ROM, ratio of means; and CI, confidence interval [26,31,32].

In the last meta-analysis, three studies on IL-6, with a total of 156 subjects in the experimental group and 158 subjects in the control group, were included (Figure 6). Results generated by the random effects model showed no statistically significant improvements in the experimental group, compared to the control group (ROM: 0.66; 95% CI: 0.39 to 1.13; *p* = 0.13). High heterogeneity between studies was observed: I2 = 99%, *p* < 0.01 (Figure 6), while no substantial report of publication bias was found [Egger’s test t = 0.32, *p* = 0.801 bias estimate 5.11 (SE = 15.8), Supplementary Figure S1, funnel plot].

### 3.4. Study Characteristics: Systematic Review of Oxidative and Inflammation Markers in Plant-Based Diet

A summary of the main features of studies comparing levels of oxidative stress (MDA, 8OHdG, and DNA damage via comet assay, GSH) and early-stage inflammation (CRP, IL-6, TNF-α) in healthy subjects following a plant-based dietary regimen generally compared to subjects including meat in their diet is shown in Table 2.

The study conducted by Boanca et al. observed that the reduced concentration of TNF-α and serum MDA in lacto-ovo vegetarians (LOVs) suggests a lower level of inflammation and oxidative stress due to a reduced level of free radicals [35]. Boanca’s results showed significantly lower levels of serum vitamin B12, erythrocyte SOD activity, and mean serum MDA in LOV individuals compared to non-vegetarians. The duration of the LOV diet was not significantly associated with serum vitamin B12 nor with MDA concentrations. However, a significant inverse correlation was found between SOD activity and the duration of adherence to the LOV diet. The conclusions suggest that only the duration of the LOV diet affects SOD activity, and further research is needed to understand the underlying molecular mechanisms [35].

Poornima et al. observed that there are no significant differences in TNF-α and MDA levels between vegetarians and fish consumers, although vegetarians showed significantly higher levels of ascorbic acid [36].

Results from Nebl et al. show that plasma MDA levels increase after physical exercise in omnivores, lacto-ovo vegetarians, and vegan subjects but with statistically significant increases only in these latter groups. Prior to physical exercise, the three groups had comparable concentrations of plasma MDA. These findings indicate that physical exercise increases oxidative stress in all three groups but with variations in MDA levels among different dietary types [37]. The study conducted by Dietrich and colleagues observed lower levels of excretion in urine of 8OHdG and 8-iso-PGF2α in vegans compared to the control group (omnivores). The vegan diet did not show to be associated with lower levels of MDA and protein carbonyls when compared to the omnivorous group [38]. In the study by Kazˇimírová et al., non-vegetarians showed higher levels of DNA strand breaks and oxidized purines compared to vegetarians. The lowest level of DNA damage (as observed by comet assay) was found in lymphocytes of lacto-ovo vegetarians, suggesting that this diet may provide some protection against oxidative stress [39]. A positive correlation between age and oxidative DNA damage was found in non-vegetarians, whereas the opposite trend was observed in vegetarians.

Verhagen et al. evaluated the effect of DNA damage using the comet assay technique on individuals following a vegan diet versus omnivore diet. The results showed that the vegan diet has the potential to reduce DNA damage and genomic instability, possibly reducing the risk of diseases related to these conditions [40]. Finally, Gajski et al. showed that DNA damage was significantly higher in vegetarian subjects compared to non-vegetarians. When dividing the subjects into specific subgroups, it emerged that a pescatarian diet, which could be defined as plant-based even if it includes an adequate intake of fish and seafood, could be a more beneficial diet for maintaining DNA integrity. Statistically significant differences were found only between vegetarians and pescatarians [41].

Focusing on the vegetarian diet, Szeto and colleagues revealed that long-term vegetarians have lower concentrations of high-sensitivity C-reactive protein (hsCRP) compared to omnivores [42]. These data suggest that a prolonged vegetarian diet may have anti-inflammatory and cardioprotective effects, as lower levels of hsCRP are associated with a reduced risk of cardiovascular diseases. Additionally, a significant inverse correlation was observed between ascorbic acid and hsCRP in apparently healthy subjects, suggesting a potential anti-inflammatory role for ascorbic acid [42]. A study conducted by Franco-de Moraes et al. observed differences in the composition of gut microbiota among individuals with different dietary lifestyles, who also appear to have modification in their inflammatory and metabolic profiles [43]. This study suggested that reducing consumption of animal-derived foods may promote a specific intestinal environment. Indeed, it was observed that individuals with a vegetarian lifestyle show significantly lower levels of CRP compared to omnivores [43]. Similarly, numerous studies showed a significant decrease in the CRP levels in vegetarians compared to omnivorous, highlighting the importance of this marker for predicting cardiovascular risk and directing therapeutic interventions [44,45,46,47,48,49,50]. In contrast, studies conducted by Yang in 2011 found no difference in hsCRP levels in vegetarian and omnivore participants [51].

Bogotaj Jontez et al. investigated individuals following low-carbohydrate high-fat, vegan, vegetarian, and omnivorous diets with a cross-sectional study. No significant differences between these groups were found [52]. CRP levels also did not differ significantly in the study published by Rowicka’ and colleagues, which did not report differences in the CRP level of prepubertal children following a vegetarian diet and those following an omnivorous diet [53]. The only differences were reported in GSH values, which improved in vegetarian people [53]. Studies by Menzel et al., 2020, and Sebeková et al., 2001 [54,55], found no significant differences in hsCRP levels between vegans and omnivores. The unadjusted model showed a tendency for omnivores to have higher hsCRP levels (0.94 mg/L) compared to vegans (0.60 mg/L), but this was not statistically significant (*p* = 0.09) [54]. Results from Sutliffe et al. showed significant changes in CRP levels among participants to an intervention study proposing a vegan diet [56]. These results indicate that a vegan diet can lead to significant reductions in CRP levels, suggesting a potential decrease in inflammation and cardiovascular risk [56,57]. The extent of CRP reduction was influenced by baseline CRP levels and varied between genders. On the other hand, Lederer et al. 2020 showed not significant value in CRP variation between vegan and omnivore subjects [58], and different studies showed no differences in IL-6 and TNF-aplha values between vegetarian and control diets [58,59,60,61].

**Table 2:** Markers of oxidative and early-stage inflammation and their impact on plant-based diet in healthy subjects.

Study, Year	Type of Study	Population	Mean, Age, Years (SD/IC)	Sex (M/F)	Dietary Pattern/Dietary Assessment Method	Biomarker	Results	*p* Value
Boanca et al., 2014 [35]	Observational study	Lacto-ovo vegetarians *n* = 48 Non-vegetarians *n* = 38	Lacto-ovo vegetarians 28.4 ± 8.6 Non-vegetarians 29.8 ± 10.1	Non-vegetarians (13 M/25 F) Lacto-ovo vegetarians (16 M/32 F)	Lacto-ovo vegetarians/Non-vegetarians Adherence to diet: not reported	Serum MDA	Lacto-ovo vegetarians 2.39 ± 1.02 Non-vegetarian 3.08 ± 1.08	<0.05
Poornima et al., 2003 [36]	NA	Vegetarians *n* = 23 Fish eaters *n* = 22	Vegetarians 45.95 ± 4.33 Fish eaters 46.05 ± 4.14	Vegetarians (23 M) Fish eaters (22 M)	Vegetarians/Fish eaters (Non-vegetarians) Adherence to diet: not reported	Plasma MDA GSH	MDA: Vegetarians 4.78 ± 2.65 Fish eaters 4.11 ± 1.93 GSH: Fish eaters 32.48 + 9.60 Control 31.72 + 7.4	NS NS
Nebl et al., 2019 [37]	Cross-sectional study	Omnivore (OMN) diet *n* = 25 Lacto-ovo vegetarian (LOV) diet *n* = 25 Vegan diet *n* = 23	OMN diet 27.2 ± 4.05 LOV diet 27.6 ± 4.31 Vegan diet 27.5 ± 4.26	OMN diet (10 M/15 F) LOV diet (10 M/15 F) Vegan diet (8 M/15 F)	Omnivore diet/Lacto-ovo diet/Vegan diet Adherence to diet: not reported	Plasma MDA	OMN diet Pre 0.52 ± 0.09/Post 0.56 ± 0.10 LOV diet Pre 0.50 ± 0.07/Post 0.62 ± 0.15 VEG diet Pre 0.57 ± 0.13/Post 0.68 ± 0.15 (uM)	<0.01
Dietrich et al., 2022 [38]	Cross-sectional study	Omnivore (OMN) diet *n* = 36 Vegan (VGN) diet *n* = 36	Omnivore diet 38.5 (32.0–46.0) Vegan diet 37.5 (32.5–44.0)	Omnivore diet (18 M/18 F) Vegan diet (18 M/18 F)	Vegan diet/Omnivore diet Adherence to diet assess by three-day dietary records	8OHdG MDA	8OHdG, nmol/d VGN 13.5 (11.6–15.7) OMN 15.8 (13.6–18.5) MDA, umol/L VGN 1.04 (0.90–1.20) OMN 1.08 (0.94–1.25)	0.04 0.73
Kažimírová et al., 2004 [39]	NA	Vegetarian diet *n* = 24 Non-vegetarian diet *n* = 24	Vegetarian diet (40.0 ± 1.7) Non-vegetarian diet (41.2 ± 2.0)	Vegetarian diet (12 M/12 F) Non-vegetarian diet (12 M/12 F)	Vegetarian diet/Non-vegetarian diet Adherence to diet: not reported	Comet Assay	Vegetarian diet 99.8 ± 7.7 Non-vegetarian diet 116.3 ± 10.5	0.017
Verhagen et al., 1996 [40]	Cross sectional study	Vegan diet *n* = 20 Omnivore diet *n* = 20	Vegan diet (46 ± 11, Range 27–69) Omnivore diet (44 ± 10, range 27–65)	Vegan diet (19 M/1 F) Omnivore diet (19 M/1 F)	Vegan diet/Omnivore dietAdherence to diet: not reported	Comet Assay	Vegan diet Tail moment 0.37 ± 0.15 Omnivore diet 0.43 ± 0.17 Tail moment	<0.05
Gajski et al., 2023 [41]	NA	Vegetarian diet *n* = 16 Non-vegetarian diet *n* = 16	Vegetarian diet 31.4 ± 3.1 Non-vegetarian diet 31.0 ± 3.5	Vegetarian Diet (16 F) Non-vegetarian diet (16 F)	Vegetarian/Non-vegetarian Adherence to diet: not reported	Comet Assay	(1) 3.6 ± 1.1 Tail intensity (2) 2.8 ± 1.0 Tail intensity	<0.05
**Franco-de Moraes et al., 2017** [43]	Case control study	Vegetarians *n* = 102 Omnivores *n* = 100	Vegetarians 49.6 ± 8.6 Omnivores 49.1 ± 8.2	(123 M/145 F)	Vegetarian/Omnivore Adherence to diet assess by food intake report (not specified)	CRP TNF-alpha	CRP Vegetarian 0.50 mg/L Omnivore 1.1 mg/L TNF-alpha Vegetarian 2.9 (IQ 1.5–5.0) Omnivore 2.9 (IQ 1.9–4.5)	0.007 0.015
**Krajcovicova-Kudlackova et al., 2005** [44]	NA	Vegetarian *n* = 133 Omnivore *n* = 137	Vegetarian 46.2 ± 1.4 Omnivore 47.2 ± 1.4	Vegetarian (51 M/86 F) Omnivore (45 M/88 F)	Vegetarian/Omnivore Adherence to diet assess by food intake report (not specified)	CRP	Vegetarian 0.72 ± 0.81 Omnivore 1.62 ± 1.40	<0.001
**Chen et al., 2008** [46]	Cross-sectional study	Vegetarian *n* = 99 Omnivore *n* = 99	Vegetarian 51.24 ± 8.88 Omnivore 49.38 ± 9.60	Vegetarian (38 M/65 F) Omnivore (53 M/46 F)	Vegetarian/Omnivore Adherence to diet assess by questionnaire of dietary preferences	CRP	Vegetarian 1.40 ± 2.30 Omnivore 2.23 ± 4.40	0.025
**Hung et al., 2008** [47]	Cross-sectional study	Vegetarian *n* = 71 Omnivore *n* = 388	Vegetarian 49.1 ± 11.2 Omnivore 50.6 ± 9.5	Vegetarian (25 M/46 F) Omnivore (240 M/148 F)	Vegetarian/Omnivore Adherence to diet assess by questionnaire regarding lifestyle	CRP	Vegetarian 1.70 ± 1.30 Omnivore 2.30 ± 3.20	0.003
Su et al., 2011 [48]	Cross-sectional study	Vegetarian *n* = 49 Omnivore *n* = 41	Vegetarians 58.6 ± 6.0 Omnivores 57.2 ± 5.4	Vegetarian (49 F) Omnivore (41 F)	Vegetarian/Omnivore Adherence to diet: vegetarians who followed diets that excluded meat, fish, and poultry for at least 5 years	CRP	Vegetarian 0.70 ± 0.70 Omnivore 0.90 ± 1.20	NS
Chuang et al., 2016 [49]	Prospective matched cohort design	Vegetarian *n* = 686 Omnivore *n* = 3423	Vegetarian 45.2 ± 12.3 Omnivore 45.1 ± 12.2	Vegetarian (186 M/500 F) Omnivore (929 M/2494 F)	Vegetarian/Omnivore Adherence to diet assess by self-administered FFQ	CRP	Vegetarian 1.60 ± 2.50 Omnivore 2.00 ± 4.60	0.061
Acosta-Navarro et al., 2017 [50]	Cross-sectional study	Vegetarian *n* = 44 Omnivore *n* = 44	Vegetarian 45.5 ± 7.8 Omnivore 46.8 ± 9.6	Vegetarian (44 M) Omnivore (44 M)	Vegetarian/Omnivore Adherence to diet assess by questionnaires regarding dietary preferences	CRP	Vegetarian 0.98 ± 0.72 Omnivore 1.47 ± 1.37	0.080
Bogataj Jontez et al., 2023 [52]	Cross-sectional study	Vegan *n* = 32 Vegetarian *n* = 37 Omnivore *n* = 37	Vegan 34.0 ± 10.1 Vegetarian 37.4 ± 10.7 Omnivore 36.2 ± 11.5	Vegan (9 M/23 F) Vegetarian (7 M/30 F) Omnivore (12 M/25 F)	Vegan/Vegetarian/Omnivore Adherence to diet assess by FFQ	CRP	Vegan 0.80 ± 1.08 Vegetarian 0.75 ± 1.01 Omnivore 1.20 ± 1.77	NS
Rowicka et al., 2023 [53]	Cross-sectional study	Vegetarian *n* = 32 Omnivore *n* = 40	Vegetarian 6.6 ± 2.5 Omnivore 6.4 ± 2.4	Vegetarian (15 M/17 F) Omnivore (18 M/22 F)	Vegetarian/Omnivore Adherence to diet assess by food diary	CRP GSH	CRP Vegetarian 0.40 Omnivore 0.30 GSH Vegetarian 6.61 Omnivore 17.03	0.190 0.001
Menzel et al., 2020 [54]	Cross-sectional study	Vegan *n* = 36 Omnivore *n* = 36	Vegan 37.5 (32.5–44.0) Omnivore 38.5 (32.0–46.0)	Vegan (18 M/18 F) Omnivore (18 M/18 F)	Vegan/Omnivore Adherence to diet: not reported	CRP	Vegan 0.60 mg/L Omnivore 0.94 mg/L	NS
Sebekova et al., 2001 [55]	NA	Vegan *n* = 9 Omnivore *n* = 19	Omnivore (30.5 ± 1.6) Vegan (39.6 ± 3)	NA	Vegetarian/Vegan/Omnivore Adherence to diet assess by food frequency questionnaire	CRP	Vegetarian 0.40 ± 1.71 Vegan 0.87 ± 1.35 Omnivore 0.81 ± 1.13	NS
Sutliffe et al., 2015 [56]	NA	Pre-interventions *n* = 604 Post-interventions *n* = 604	Pre and post 58.0 ± 15.4	Pre and post (196 M/408 F)	Vegan Adherence to diet: self-reported	CRP	Pre 5.2 ± 0.5 Post 3.7 ± 0.4	<0.001
Jenko Pražnikar et al., 2023 [57]	NA	Vegan *n* = 24 Vegetarian *n* = 21 Omnivore *n* = 24	Vegan 33.6 ± 9.6 Vegetarian 37.1 ± 10.8 Omnivore 36.2 ± 10.4	Vegan % (33.3 M/66.7 F) Vegetarian % (33.3 M/66.7 F) Omnivore % (33.3 M/66.7 F)	Vegan/Vegetarian/Omnivore Adherence to diet assess by FFQ	CRPTNF-alpha IL-6	CRP Vegan 0.34 ± 0.66 Vegetarian 0.31 ± 0.80 Omnivore 0.37 ± 2.01 TNF-alpha Vegetarian 0.57 (0.64) Omnivore 0.37 (0.27) IL-6 Vegetarian diet 1.22 (0.59) Omnivore diet 1.30 (2.80)	NS NS NS
Lederer et al., 2022 [58]	Randomized controlled trial	Vegan *n* = 26 Meat-rich *n* = 27	Vegan 33.2 ± 11.2 Meat-rich 29.9 ± 9.5	Vegan (69 F/31 M) Meat-rich (56 F/44 M)	Vegan/Meat-rich Adherence to diet assess according to guidelines of German nutrition association	CRP	Vegan 1.3 ± 1.5 Meat-rich 1.4 ± 1.7	NS
Sofi et al., 2018 [59]	Randomized trial	Vegetarian diet *n* = 60 Mediterranean diet *n* = 58	Vegetarian diet 49.5 (24–70) Mediterranean diet 52 (21–75)	Vegetarian diet (11 M/49 F) Mediterranean diet (15 M/43 F)	Vegetarian diet/Mediterranean diet Adherence to diet assess by one-week diary	TNF-alpha	Vegetarian diet 3.50 Mediterranean diet 2.26	NS
Sebekova et al., 2006 [62]	Cross-sectional study	Vegetarian *n* = 90 Omnivore *n* = 46	Vegetarian 37.7 (35.1–40.3) Omnivore 37.1 (33.5–40.7)	Vegetarian (30 M/60 F) Omnivore (19 M/27 F)	Vegetarian/Omnivore Adherence to diet assess by food frequency questionnaire	CRP	Vegetarian 0.87 ± 1.38 Omnivore 0.81 ± 0.97	NS
Chen et al., 2011 [63]	Cross-sectional study	Vegetarian *n* = 173 Omnivore *n* = 190	Vegetarians 49.94 ± 9.77 Omnivores 54.00 ± 9.70	Vegetarian (173 F) Omnivore (190 F)	Vegetarian/Omnivore Adherence to diet assess by questionnaire regarding lifestyle; their dietary preferences	CRP	Vegetarian 1.80 ± 3.40 Omnivore 1.20 ± 1.80	0.05
Lee et al., 2014 [64]	Cross-sectional study	Vegetarian *n* = 357 Omnivore *n* = 357	Vegetarians 52.8 ± 8.5 Omnivores 53.8 ± 8.7	Vegetarian (156 M/201) Omnivore (156 M/201)	Vegetarian/Omnivore Adherence to diet: subjects who answered ‘‘yes’’ to the question ‘‘Are you vegetarian?’’	CRP	Vegetarian 1 ± 2 Omnivore 1 ± 2	NS
Flagg et al., 1993 [65]	NA	Vegetarian *n* = 6 Non-vegetarian *n* = 6	Range 18–60	NA	Vegetarian/Non-vegetarian Adherence to diet assess by HHQ	GSH	Vegetarian 1440 Non-vegetarian 773	0.002

FFQ, food frequency questionnaire; and HHQ, Health Habits and History questionnaire.

### 3.5. Meta-Analysis of Oxidative and Inflammation Markers in Plant-Based Diet

**Figure 7:**
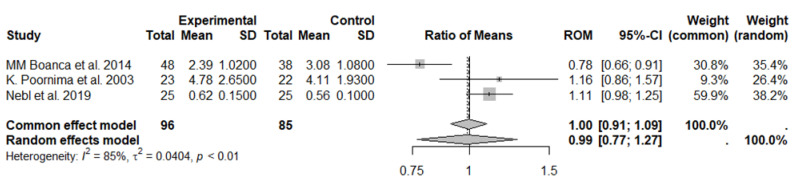
Forest plot showing the effects of vegetarian diet on MDA. SD, standard deviation; ROM, ratio of means; and CI, confidence interval [35,36,37].

In the first meta-analysis, three studies on MDA, with a total of 96 subjects in the experimental group (vegetarian diet) and 85 subjects in the control group (control diet), were included (Figure 7). Results generated by the random effects model showed no statistically significant differences between the study groups (ROM: 0.99; 95% CI: 0.77 to 1.27; *p* = 0.9310). High heterogeneity between studies was observed: I^2^ = 85%, *p* < 0.01 (Figure 7), and no evidence of publication bias was suggested by the funnel plot and the Egger’s test (t = −0.11, *p* = 0.928 bias estimate −0.70 (SE = 6.2)).

**Figure 8:**
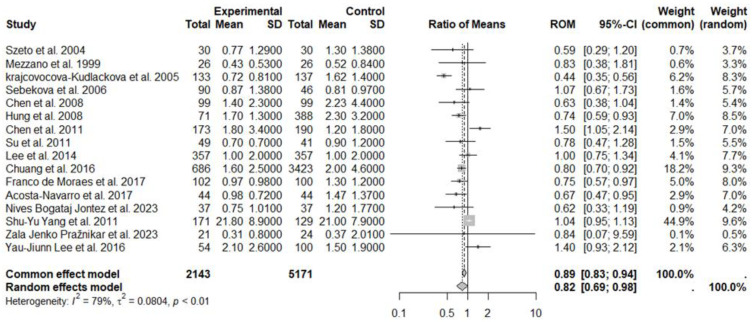
Forest plot showing the effects of vegetarian diet on CRP. SD, standard deviation; ROM, ratio of means; and CI, confidence interval [42,43,44,45,46,47,48,49,50,51,52,57,61,62,63,64].

In the second meta-analysis, three studies on CRP, with a total of 2143 subjects following a vegetarian diet and 5171 subjects in the control group (control diet), were included (Figure 8). Results generated by the random effects model showed in vegetarians a 20% reduction in CRP levels compared to the control group (ROM: 0.82; 95% CI: 0.69 to 0.98; *p* = 0.0297), despite the high heterogeneity between studies: I^2^ = 79%, *p* < 0.01 (Figure 8). No evidence of publication bias was found (Egger’s test t = −1.04, *p* = 0.316 bias estimate −0.93 (SE = 0.89)). The sensitivity analysis showed that by omitting Hung et al. 2008 [47] (ROM = 0.83, *p* = 0.06) or omitting Acosta-Navarro et al. 2017 [50] (ROM = 0.84, *p* = 0.06), the effect size was not statistically significant between the study groups.

**Figure 9:**
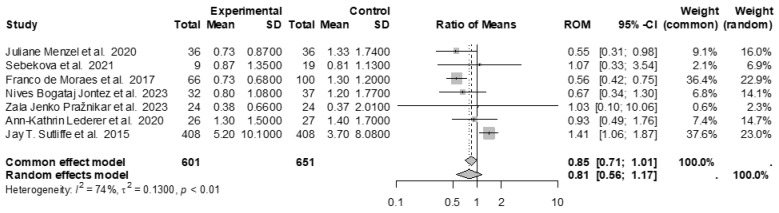
Forest plot showing the effects of vegan diet on CRP. SD, standard deviation; ROM, ratio of means; and CI, confidence interval [43,52,54,55,56,57,66].

Similar studies on CRP, comparing vegan diet to controls, have been evaluated in an independent meta-analysis. Seven studies, with a total of 601 subjects in the experimental group (vegan diet) and 651 subjects in the control group (control diet), were included (Figure 8). No significant difference between groups was found by the fixed or random models (ROM: 0.81; 95% CI: 0.56 to 1.17; *p* = 0.2544). The results were affected by high heterogeneity between studies (I^2^ = 74%, *p* < 0.01) (Figure 9), with no evidence of publication bias (Egger’s test t = −0.20, *p* = 0.846 bias estimate −0.32 (SE = 1.57)). The sensitivity analysis showed that by omitting Jay T. Sutliffe et al. 2015 [56] (ROM = 0.62, *p* < 0.0001), the effect size was statistically different between the study groups.

**Figure 10:**
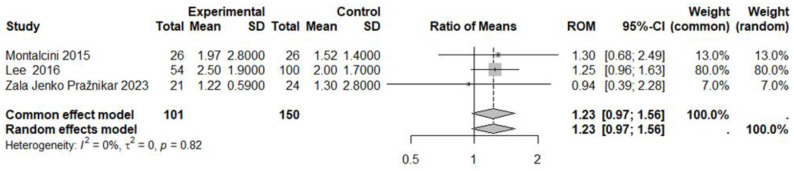
Forest plot showing the effects of vegetarian diet on IL-6. SD, standard deviation; ROM, ratio of means; and CI, confidence interval [57,60,61].

The fourth meta-analysis on IL-6 included three studies with a total of 101 subjects in the experimental group (vegetarian diet) and 150 subjects in the control group (control diet) (Figure 10). No statistically significant effect was observed in the experimental group in comparison to the control group (ROM: 1.23; 95% CI: 0.97 to 1.55; *p* = 0.08). The heterogeneity was low (I^2^ = 0%, *p* = 0.82) (Figure 10), and the publication bias was absent [Egger’s test t = −0.81, *p* = 0.567 bias estimate −0.477 (SE = 0.59), Supplementary Figure S2, funnel plot)].

The sources of heterogeneity were investigated including meta-regression models with terms for age, sample size, and adherence assessment. None of these items turned out to significantly explain heterogeneity. Only for the analysis regarding CRP in the vegan diet did heterogeneity account for 2.4% of the variability. Furthermore, if different types of diets, such as vegan versus omnivorous, are compared, this category has an R-squared of 100%, fully explaining the heterogeneity.

## 4. Discussion

This systematic review and meta-analysis examined the effects of the Mediterranean, vegetarian, and vegan diets on biomarkers of oxidative stress (such as MDA and 8OHdG) and early-stage inflammation (such as CRP, IL-6, and TNF-α), with multiple aims, including the identification of these biomarkers in the pathogenesis of diet-related diseases, a possible ranking of biomarkers based on their sensitivity to different diets, and especially a comparison of different dietary regimens according to their ability to reduce oxidative stress and inflammation. These aims can be summarized with the identification of most effective strategies to reduce the risk of chronic diseases linked to inflammation and oxidative stress.

These diets were selected from a broad range of dietary regimens because they are the most extensively studied in the scientific literature on oxidative stress and inflammation, particularly in populations without underlying diseases. MedDiet is a dietary pattern characterized by a high consumption of whole foods, vegetables, fruits, legumes, grains, nuts, olive oil as a principal source of fat, a limited intake of meat, and moderate consumption of dairy and alcohol intake during meals. Olive oil is predominant in the MedDiet, while saturated fats are present in small amounts. This dietary pattern allows for higher intake of omega-3 fatty acids and monounsaturated fatty acids and a reduction in carbohydrates, particularly simple sugars, such as those in sweets and refined foods. The MedDiet can be considered a cornerstone in preventive medicine, particularly for diseases related to an imbalance in oxidative levels. Its effectiveness stems from the combination of various foods with antioxidant and anti-inflammatory effects [24]. Similarly, the vegetarian lifestyle is generally considered healthy, and differences in biomarkers related to diseases such as cancer might be expected between vegetarians and non-vegetarians [39]. This diet is characteristically rich in carotenoids and maintains higher antioxidant vitamin status (vitamins C and E), although other components such as zinc and vitamin B12 may be deficient and therefore require attention [35]. Among plant-based diets, the vegan diet, which is rich in fresh fruits, vegetables, whole grains, legumes, nuts, and seeds, has been shown to significantly reduce systemic inflammation with variations observed based on gender and baseline health conditions [4].

Diets rich in fruits and vegetables are believed to protect against chronic and degenerative diseases, including cardiovascular diseases, type 2 diabetes, types of cancer, and cognitive decline [67,68]. This protective effect is attributed to antioxidant compounds of plant origin such as ascorbic acid, carotenoids, and flavonoids. These antioxidants have the potential to protect important biological sites, including lipoproteins, cell membranes, and DNA, from oxidative damage [42]. Studies indicate that a vegetarian diet, when followed correctly, can provide adequate nutrition and offer health benefits, particularly in reducing the risk of chronic disease [69,70,71,72]. Recent evidence suggests that plant-based diets can influence inflammatory biomarker profiles, leading to a reduction [36]. Plant-based diets are also becoming increasingly popular, both for their potential in preventing diseases and also for their positive impact on the environment [71].

On the other hand, it is important to show how an unbalanced diet and an unhealthy lifestyle are associated with elevated levels of oxidative stress biomarkers, promoting the development of diseases [73,74]. Indeed, elevated concentrations of ROS or RNS can lead to oxidative stress and damage vital biomolecules such as DNA, proteins, and membranes [75]. The unbalanced production of hydroxyl radicals and peroxynitrite can induce lipid peroxidation, thus damaging cellular membranes and lipoproteins [76]. As a result, MDA and 4-hydroxy-2-nonenal are formed, both recognized as cytotoxic and mutagenic [77]. Prolonged oxidative stress induces DNA lesions, forming 8OHdG, identified as a tissue biomarker of oxidative stress, associated with carcinogenesis, accelerated aging, and mutagenesis [78,79]. Furthermore, inflammatory processes are closely related to oxidative stress and may influence the concentrations of oxidative stress biomarkers. Increased oxidative stress linked to inflammatory processes results in a persistent loop of DNA damage and subsequent repair, thus driving a higher rate of cell turnover and increasing the possibility of genetic errors [80,81].

Oxidative stress can be prevented by antioxidants, which function as free radical scavengers or as inducers of endogenous antioxidant systems and therefore as an ROS antagonist.

Nrf2 and NF-*κ*B are essential in the cellular defense against oxidative stress and inflammatory responses. Recent evidence shows that the Nrf2 signaling pathway interacts with the NF-*κ*B pathway during oxidative stress. Notably, numerous natural compounds have been widely acknowledged for their capacity to mitigate oxidative stress and inflammation by modulating the Nrf2 and/or NF-κB signaling pathways. Molecular studies emphasized the interaction between Nrf2 and NF-κB, demonstrating its involvement in the pathophysiology of various diseases. A variety of dietary compounds can activate the Nrf2 signaling pathway, boosting the expression of Nrf2 and its target genes while enhancing its nuclear translocation to promote antioxidant activity [82]. According to our best knowledge, only one study analyzed NF-kB levels in 11 women aged 27, following either a Mediterranean or Western diet, but specifically evaluated postprandial serum levels. The authors did not report any differences between the two diets. According to our best knowledge, only one study analyzed NF-kB levels in 11 women aged 27.5 ± 6.55 years with normal weight (BMI: 21.8 ± 1.56), following either a Mediterranean or Western diet, but specifically evaluated postprandial serum levels. The authors did not report any differences between the two diets [83].

Antioxidants in diets are components of the antioxidant defense system and can therefore help reduce ROS concentrations [73]. However, data on antioxidant levels in diets are contradictory, with varying antioxidant levels depending on the type of diet [31]. Considering this scenario, the study aimed to examine the effect of MedDiet and plant-based diets on biomarkers of oxidative stress and early-stage inflammation in healthy subjects, evaluating whether dietary regimens could represent a lifestyle intervention suitable for preventing or improving conditions associated with the pathogenesis of chronic diseases or improving patient recovery process in rehabilitation.

Results obtained from the meta-analysis regarding the MedDiet showed an improvement in the experimental groups compared to control diets, although the results generally lacked statistical significance. Specifically, we observed reduction in MDA (ROM: 0.80; 95% CI: 0.57 to 1.13; *p* = 0.2092), 8OHdG (ROM: 0.81; 95% CI: 0.59 to 1.11; *p* = 0.1847), CRP (ROM: 0.72; 95% CI: 0.42 to 1.23; *p* = 0.1545), and IL-6 (ROM: 1.23; 95% CI: 0.97 to 1.55; *p* = 0.08) in individuals following the MedDiet. This result might be influenced by the high heterogeneity of the studies reflecting variations in participant factors, such as genetic differences, lifestyle habits, and adherence levels to the diet, not to speak about the variability of the control groups.

**Figure 11:**
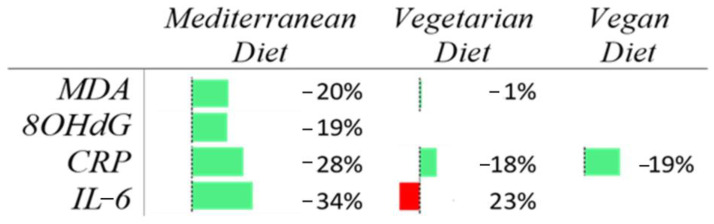
Schema of the results of the meta-analysis. The green color indicates the positive effect of the diet on the biomarker of oxidative stress or early-stage inflammation. The red color indicates the negative effect of the diet on the biomarkes of early-stage of inflammation. Empty cells are those where we were unable to perform the meta-analysis.

However, results from most studies suggested that the MedDiet might provide long-term protective effects. The vegetarian diet showed significant reductions in CRP levels (ROM: 0.82; 95% CI: 0.69 to 0.98; *p* = 0.0297) compared to omnivorous diets, suggesting a correlation between this diet and decreased systemic inflammation. In contrast, a vegan diet did not show a clear reduction in the early biomarker of inflammation compared to omnivorous diets, although non-significant improvements in CRP levels were noted (ROM: 0.81; 95% CI: 0.56 to 1.17; *p* = 0.2544). Similarly, the effects on MDA (ROM: 0.99; 95% CI: 0.77 to 1.27; *p* = 0.9310) and IL-6 (ROM: 1.23; 95% CI: 0.97 to 1.55; *p* = 0.08) were not significant. A summary of results from the meta-analyses evaluating the effect of different dietary regimens on the selected biomarkers of oxidative stress and early inflammation is reported in Figure 11.

The results shown in Figure 11 show clearly that both MedDiet and plant-based diets are likely to reduce the level of the most commonly investigated biomarkers of oxidative stress or early inflammation. The reliability of these estimates is limited by the high heterogeneity across studies, arising from differences in dietary program duration and biomarker measurement methods. In addition, the small size of studies evaluated resulted in most studies being statistically non-significant. Although, it is evident that most estimates point towards an improvement in oxidative stress or early-stage groups randomized to mediterranean, vegetarian, and vegan diets. No clear ranking appears among biomarkers, although biomarkers associated with inflammation show higher reduction in MedDiet, while the lack of data does not allow for any conclusions to be reached in those subjects following vegetarian or, especially, vegan diets. Another important outcome of this study is highlighting evidence for additional studies, confirming these observations and clarifying the mechanisms underlying the benefits of these dietary patterns. Such insights could greatly enhance the effectiveness of dietary preventive strategies. Finally, while this systematic review focuses on healthy individuals, it is important to consider diet in the context of rehabilitative therapy following chronic non-communicable diseases. For example, the guidelines by Visseren et al. (2021) [83] recommend that patients recovering from myocardial infarction engage in comprehensive cardiac rehabilitation programs, which typically include dietary counseling, behavioral support, and exercise. However, the impact of these programs on adherence to a Mediterranean lifestyle remains unclear.

Recent research has shown that adherence to a Mediterranean lifestyle is linked to improved glucometabolic and lipid profiles in post-myocardial infarction patients [84]. Additionally, our previous study indicated that patients with chronic obstructive pulmonary disease (COPD) who consumed higher amounts of vegetables demonstrated a more effective rehabilitative response [5].

## 5. Limitations

Studies on other biomarkers of oxidative stress and early-stage inflammation did not meet the inclusion criteria, and those we included, such as GSH, TNF-α, and the extent of DNA damage, were too few to allow for a quantification of the results. This analysis clearly highlights the limited number of articles currently available that investigate the mechanisms that explain epidemiological results. Interestingly, the mantra reported by several papers, inviting to increase the quantity of polyphenols, seems lacking adequate mechanistic evidence. Another major drawback of the literature is the limited number of studies in healthy subjects, often favoring the use of dietary regimen as a treatment rather than a powerful tool for prevention.

The results of this study show clearly the usefulness of MedDiet or plant-based diets for contrasting oxidative stress and in turn its adverse effect on health. However, we are not yet able to determine with certainty which diet, among the Mediterranean, vegetarian, or vegan ones, is more effective in disease prevention, particularly, in which lays behind stress parameters reported, especially for the MedDiet. The antioxidant effect of these diets is lower than expected according to epidemiological results, leaving space for alternative pathways that can implement the oxidative hypothesis.

Finally, the different dietary assessment methods used by various authors introduce bias to the analysis, as they do not clearly indicate adherence to a specified diet. This is not a causative bias; on the contrary, the lack of uniformity in assessing diet adherence could lead to an underestimation of the effect caused by the diet itself and, consequently, its effects on the biomarkers considered. The same applies to the observation that the diet duration varied from 5 days to 5 years, which could similarly result in an underestimation of the diet’s effect.

## 6. Conclusions

Although all three diets show potential in reducing oxidative stress and inflammation, the antioxidant effects—particularly for the Mediterranean diet—are lower than expected, suggesting alternative mechanisms. However, it is important to emphasize that even small changes in biomarkers can be clinically significant in the long term. Further research is needed to confirm these findings and better understand the underlying mechanisms to enhance preventive health strategies.

The findings suggest that poor adherence to the Mediterranean lifestyle can be improved through comprehensive cardiac rehabilitation programs, where dietary counseling is a key element of lifestyle interventions. While further studies are needed, improving dietary habits could play a crucial role in optimizing rehabilitation outcomes.

## Data Availability

Data are contained within the article.

## Supplementary Materials

The following supporting information can be downloaded at https://www.mdpi.com/article/10.3390/nu17030548/s1, Figure S1: Funnel plot of mediterranean diet and IL-6; Figure S2: Funnel plot of vegetarian diet and IL-6.

## Registration

The protocol is registered by Prospero n. CRD42025635326.
