# Supplementary material for: Dietary Patterns, Oxidative Stress, and Early Inflammation: A Systematic Review and Meta-Analysis Comparing Mediterranean, Vegan, and Vegetarian Diets
Source: Nutrients. 2025 Jan 31;17(3):548. doi: 10.3390/nu17030548 (PMC11819869; doi:10.3390/nu17030548)
Supplement: Supplementary file 1 [file nutrients-17-00548-s001.zip › nutrients-3428068-supplementary.pdf]

Supplemetary figures

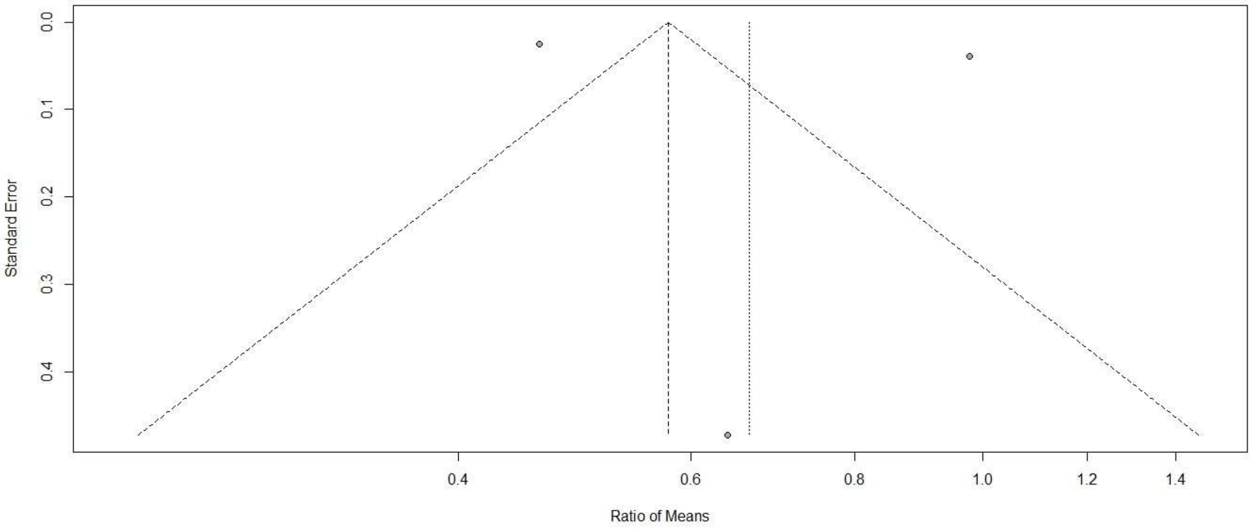

Figure S1: Funnel plot of mediterranean diet and IL6

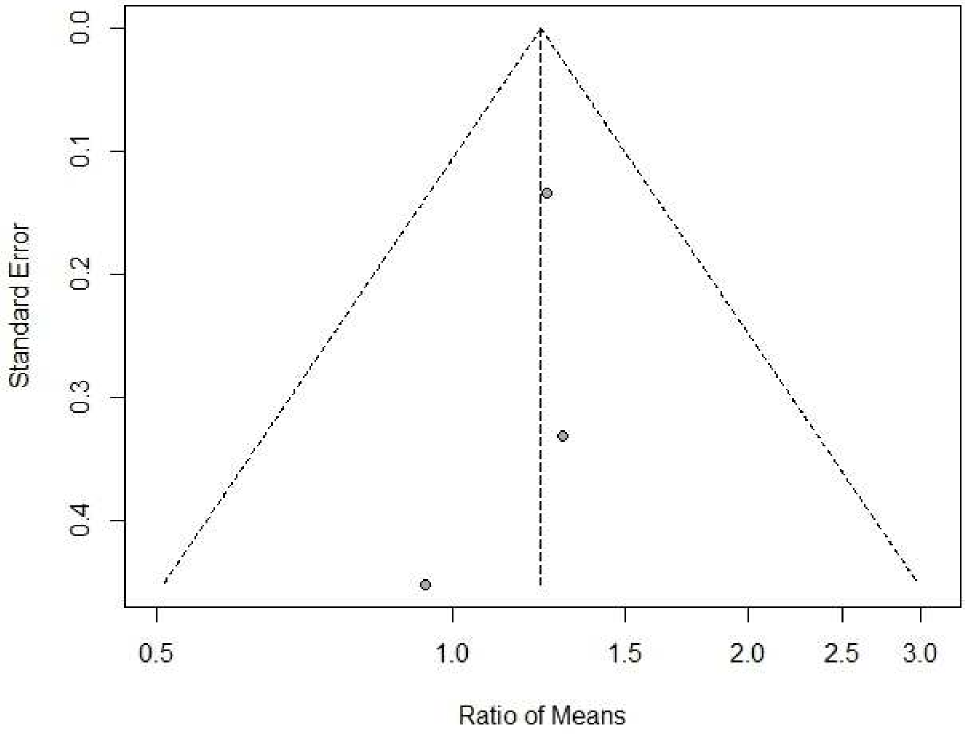

Figure S2: Funnel plot of vegetarian diet and IL6
